# Oral Health Workforce and American Indian and Alaska Native Communities: a Systematic Review

**DOI:** 10.1007/s40615-023-01515-7

**Published:** 2023-01-24

**Authors:** Amanda J. Llaneza, Julie Seward, Alex Holt, Lancer D. Stephens

**Affiliations:** 1 Southern Plains Tribal Health Board, Oklahoma City, OK USA; 2 Health Promotion Sciences, Hudson College of Public Health, University of Oklahoma Health Sciences Center, Oklahoma City, OK USA; 3 Oklahoma Shared Clinical and Translational Resources, University of Oklahoma Health Sciences Center, Oklahoma City, OK USA

**Keywords:** Oral health, Oral epidemiology, Community dentistry, American Indian/Alaska Native

## Abstract

**Objective:**

Understanding the oral health workforce representing and serving American Indian and Alaska Native (AI/AN) communities is vital to improving community dental health outcomes. No systematic review of recent published literature on the oral health workforce among this population has been completed.

**Methods:**

We conducted a systematic review of published literature examining the oral health workforce representing and serving AI/AN communities in the USA. We analyzed 12 articles according to the PRISMA Statement.

**Results:**

The studies suggested that AI/AN identity is an important aspect of routine and accessible oral healthcare. There are unique barriers and motivations that personnel in the oral health workforce face, let alone the distinctiveness of serving AI/AN communities.

**Conclusions:**

This review provides evidence that expanded oral health positions aid in community members receiving more routine and preventative care and is an upstream public health approach that has diversified the dental workforce.

## Introduction

The oral health care delivery system for American Indians/Alaska Natives (AI/AN) is complex and can be arduous to access as a patient. The nature of these complexities produces wicked problems that call for re-evaluation of the current structures and systems in place that can lead to innovative solutions. While numerous initiatives and interventions have improved the oral health status of AI/ANs, disparities continue to exist. These disparities led the authors to take a closer look at the oral health workforce, one vital factor in improved outcomes. Recent statistics from the US Census Bureau show the total AI/AN alone and in combination population increased from 5.2 million in 2010 to 9.7 million in 2020 indicating an 86.5% increase resulting in AI/AN now representing 2.9% of the US population [1]. Even prior to the increase in the AI/AN population, AI/AN are not represented proportionately in the oral health workforce and there are a lack of providers serving this population contributing to disproportionately poorer oral health outcomes compared to the general US population. The National Institute of Dental and Craniofacial Research published a call to action in December 2021 that to strengthen the oral health workforce, diversifying the nation’s oral health professionals is a key component [2]. To better understand the amount of research that has been done in this area, and subsequently additional research that might be needed, there is a need to systematically evaluate and analyze the published literature on the oral health workforce representing and serving AI/AN communities in the USA (Table 1).

**Table 1:** Table of selected articles

Study	First author	Title	Year published	Study setting
1	Aziz, SR [3]	Racial diversity in American oral and maxillofacial surgery	2010	Nationwide
2	Bolin, KA [4]	Assessment of treatment provided by dental health aide therapists in Alaska	2008	Alaska
3	Brown, LJ [5]	Racial/ethnic variations of practicing dentists	2000	Nationwide
4	Chi, DL [6]	Dental therapists linked to improved dental outcomes for Alaska Native communities in the Yukon-Kuskokwim Delta	2018	Alaska
5	Chi, DL [7]	Provider and community perspectives of dental therapists in Alaska’s Yukon-Kuskokwim Delta: a qualitative programme evaluation	2019	Alaska
6	Chi, DL [8]	Supply of care by dental therapists and emergency dental consultations in Alaska Native communities in the Yukon-Kuskokwim delta: a mixed methods evaluation	2020	Alaska
7	Mertz, E [9]	The American Indian and Alaska Native dentist workforce in the United States	2017	Nationwide
8	Mertz, E [10]	Underrepresented minority dentists: quantifying their numbers and characterizing the communities they serve	2016	Nationwide
9	Murphy, KL [11]	Interprofessional oral health initiative in a nondental, American Indian setting	2017	IHS pediatric clinic in the northwestern USA
10	Randal, CL [12]	Organizational readiness to implement system changes in an Alaskan tribal dental care organization	2020	Alaska
11	Senturia, K [13]	Dental health aides in Alaska: a qualitative assessment to improve pediatric oral health in remote rural villages	2018	Alaska
12	Wetterhall, S [14]	Cultural context in the effort to improve oral health among Alaska Native people: the dental health aide therapist model	2011	Alaska

## Methods

Article screening and data extraction were performed by one reviewer (AJL). Assessment of the articles in regard to eligibility criteria was done by one reviewer (AJL) and confirmed by two other reviewers (AH and JS). Any inconsistencies were discussed and resolved. For all included studies, the following were extracted: bibliographic information, study design, exposure(s), and outcome(s), including definitions, characteristics of study participants, numerical results: number of participants per group, number with outcome(s), effect estimates (adjusted and unadjusted), and their standard errors. Regarding effect estimates, this included frequencies and percent for categorical variables and means, and standard deviations for continuous variables.

This review considered all English-language studies that involved human subjects in the oral health workforce representing and serving AI/AN in the USA. Exposures of interest that were considered are AI/AN communities receiving dental services in the USA. The primary outcomes of interest were type of dental workforce position/title, management, and motivators/barriers faced by practitioners. The review considered observational studies including cohort studies, case control studies, and cross-sectional studies.

The search strategy was designed to access published articles. Terms identified and their respective synonyms were used by corresponding databases, and were used in an extensive search of the literature. Full copies of articles identified by the search, and considered to meet the inclusion criteria, based on their title, abstract, and subject descriptors were obtained for data synthesis.

The databases used for this search included Ovid MEDLINE, PubMed, and Web of Science, with the searches taking place from March 2021 to December 2021. The search terms used included (oral health workforce OR dental workforce) AND (American Indian, Native American, AI/AN, Indigenous). After assessment of eligibility, the articles were qualitatively assessed. The importance of the results in terms of clinical and public health relevance are further discussed.

### Eligibility Criteria

**Fig. 1:**
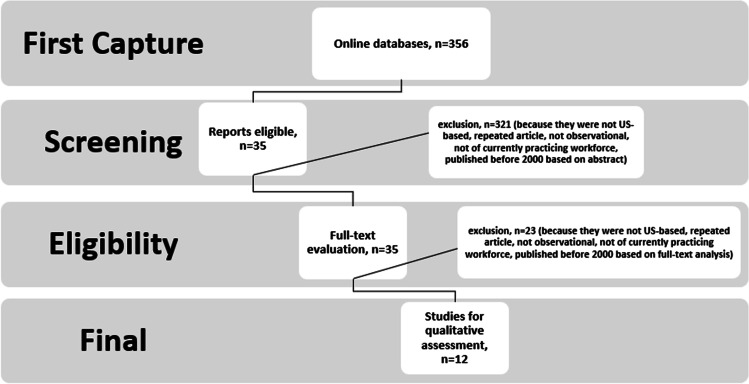
Study selection flow diagram, *N* = 12

The inclusion of articles can be seen in the diagram in Fig. 1. The eligibility criteria for this literature search were as follows:

- English-language studies published between 2000 and 2020
- Human subjects
- Observational studies only
- US location
- Currently practicing AI/AN oral health workforce representation

Inclusion of articles started with 356 articles from electronic databases using key words. Of 356 articles, 321 were excluded because they were not US-based, repeated article, not observational, not of currently practicing workforce, and published before 2000 based on the abstract. From there, 35 were included for full-text analysis. Of 35 articles, 23 were excluded because they were not US-based, repeated article, not observational, not of currently practicing workforce, and published before 2000 based on full-text analysis. The final number that met eligibility were 12 articles.

### Characteristics of Selected Studies

A total of 12 articles were analyzed for this review according to the PRISMA Statement. From the articles included, 7 articles were focused on Alaskan communities, and 5 articles included a nation-wide scope. Only 4 articles were dentist or oral surgeon-specific, 2 were Dental Health Aide Therapists (DHATS) and Primary Dental Health Aides (PDHAs) specific, and 5 included a variety of professions including dentists, DHATs, hygienists, dental assistants, and pediatric primary care physicians.

## Results

Major barriers faced by oral health workforce members included burnout, job stress, limited staffing, and lack of AI/AN representation in the workforce. One study reviewed the racial demographics of American academic oral and maxillofacial surgery positions in the USA [3]. This investigation included active dentists or oral surgeons, and of the 349 full-time faculty respondents, there were no full-time faculty of AI/AN descent sampled [3]. Furthermore, the investigators analyzed a separate survey from the American Dental Association (ADA) from 2007 to 2008 because their survey lacked representation and found that of the 9426 dental school faculty, 22 (0.2%) were American Indian/Alaska Native [3].

Amongst community members, barriers included lack of access to routine care, lack of oral health education, and lack of awareness of expanded workforce positions especially among rural communities [11, 13]. Lack of awareness of expanded workforce positions indicates that community members are not aware of the skillset such positions have in dental offices, meaning that expanded workforce position members may be seeing and caring for patients less often than expected.

Among expanded workforce positions, such as dental health aides (DHA), barriers faced include part-time employee positions with variable work hours and irregularity of hours [7, 13]. Furthermore, the irregularity of hours may be due to the lack of control they have on the frequency and duration of the visit from a visiting dentist that they support, especially in rural facilities [13]. As well, visiting general and pediatric dentists may not be familiar with the skillset of members of the expanded workforce position, underutilizing their services and not providing sufficient monitoring [13].

Among other positions such as PDHAs and DHATs, barriers include expectations of being available outside of working hours resulting in job stress and burnout [13]. A unique barrier noted is that community members are not aware of the skills and availability of expanded workforce positions, resulting in underutilization of their services [13]. Barriers specifically related to long-term retention of dental health aide therapists included not being able to work in their home community, feeling isolated, lack of social support, heavy travel schedule, and absence of childcare [7].

Major facilitators to oral health workforce positions included ownership of dental practices for dentists, local job opportunities for expanded workforce positions, service to one’s own racial/ethnic group, and services to vulnerable/low-income populations. Among practicing underrepresented minority dentists (including AI/AN, Black, and Hispanic or Latino dentists), 53.7% indicated that service to one’s own racial/ethnicity group and 58.2% reported service to vulnerable/low-income populations influenced job satisfaction [10]. Among clinically active dentists, the average percent of the AI/AN patient population treated by AI/AN dentists was 20.4%, compared to 3.7% of the AI/AN patient population treated by Black dentists, and 3.9% of the AI/AN patient population treated by Hispanic or Latino dentists. A separate study found that 67.3% of Native dentists said they were the original owners of their current primary practice, 83% of Native dentists described their current employment status as an owner, and 59.1% of Native dentists said they were very satisfied with their choice of profession [5]. To further indicate job satisfaction among currently practicing AI/AN dentists, only 8% of Native dentists indicated they were not busy enough [5].

The implementation of expanded dental workforce positions aids in addressing the challenge of shortages of dentists and access to dental care. Within AI/AN communities, training local residents as PDHAs and DHATs result in a sustainable and culturally competent solution to filling necessary workforce positions. One study found Native PDHAs and DHATs employed at Alaskan clinics understand the traditional practices of the communities which increases the trust of the community in the clinician and establishing long-term relationships with patients [13]. A cross-sectional observational study that assessed organizational readiness to implement changes to delivery of evidence-based dental care within an Alaskan tribal health care organization found organizational readiness to implement change is moderately high in a tribal health care organization for which AI/AN people work [12]. Furthermore, perceived quality of management was the only significant predictor of change commitment and change efficacy after adjusting for other factors [12].

Dental care utilization may increase when community members are part of the healthcare team. Brown et al. indicated the race/ethnicity of dentists may influence the race/ethnicity of patients who come for treatment [5]. Among the AI/AN dentists surveyed, they primarily treated white patients, followed with a relatively equal percentage of Hispanic, AI/AN and Black patients [5]. A second study investigating AI/AN dentists only in the USA found that AI/AN dentists are located in counties where AI/ANs average 8% of the population and AI/ANs make up 20.4% of their patient panels on average [10]. In regard to Census Division, Mertz et al. reported that AI/AN dentists were 1.5 times more likely to locate their practices in the Mountain Division and 2.9 times more likely in the West South-Central Division [9]. Consideration as members of the community was an important factor for expanded workforce positions as well. Providers from six Yukon Delta communities were part of a qualitative program evaluation of dental therapy [7]. Providers indicated communities accepted DHATS because of the benefits of having a Yup’ik provider who had cultural knowledge when providing care, benefit of local employment, and career opportunities within the community [7].

One study population took place among an Alaskan tribal health care organization in both urban and rural clinic settings. In urban settings, there were 24 (68.6%) providers, 20 (71.4%) were dental assistants, and 8 (53.3%) were support staff, and in rural clinic settings, there were 10 (28.6%) providers, 7 (25%) dental assistants, and 7 (46.7%) support staff [12]. In one study of the clinical dentists who reported (*n* = 329) working in expanded function, 70.0% (*n* = 230) reported the state they practice in allows employment of expanded function positions [9]. Dentists in safety-net settings were more likely to employ expanded function staff (67.8% vs 45.8%) compared to dentists in traditional settings. Traditional practices were characterized as those where practice settings were a solo practice, associate, contractor, or group practice. Safety-net practices were characterized by a practice setting within the IHS, civil hire on Indian Land, health center, hospital, armed forces, or a prison. Furthermore, the average AI/AN dentist’s clinical setting has 3 dentists, 2 registered dental hygienists, and 5 operatories and only 2% (4) of AI/AN dentists sampled for the survey employ a dental therapist [9].

In Alaskan communities, oral health positions such as DHATS and PDHAs have become an integral part of receiving oral health care. Due to the remoteness and rurality of Native Alaskan communities and villages, many dentists travel several times a year to these communities. This has created a dissonance between the dentists and community members, and due to the lack of available services, when a dentist is present, it is for major dental care such as extractions. The introduction of DHAT and PDHA positions has allowed for community members to receive more routine and preventative care. In addition, cultural barriers between dental professionals and community members have decreased resulting in increased trust and access to care for community members. Dental Health Aide Therapists are an upstream approach and have diversified the dental workforce [6, 8, 13]. This has created opportunities for community members to serve as healers and removed cultural barriers to care [6, 8, 13]. As well, increased DHAT treatment days were positively associated with child and adult preventative care and negatively associated with extractions for children and adults [6]. After dental therapy implementation, providers from six Yukon Delta communities noticed improvements in oral health in the communities in which dental therapy provided care [7]. Furthermore, there were fewer patients with large cavities and some children with no cavities because access to care improved with a dental therapist position [7]. Expanded workforce positions not only positively impacted dental health care facilities but improved prevention oriented educational efforts in schools, and the creation of a local screening and triage system in communities with a DHAT [7].

There have been concerns over the care received from expanded workforce positions; however, this should be refuted. A study investigating residents’ satisfaction with the DHAT model found that satisfaction with dental care was good and generally comparable among respondents who received care from DHATS and those treated by another provider such as general dentists or oral surgeons [14]. Furthermore, the majority of caregivers had positive responses about DHATs’ communication skills and chairside manner, and adults who participated (111) had similar responses of the children’s survey results in regard to satisfaction [14]. Many patients felt less fearful of dental procedures because DHATs are often members of the community or frequently visit the villages and may even have relatives where they are practicing [14]. Another study found no statistically significant difference (*p* = 0.77) in the number of any kind of complications resulting from the treatments provided by the DHAT members and the dentist group, separately [4]. The authors defined complications requiring further treatment as an event that requires a return visit, postoperative medications or interventions by a general dentist or specialist [4]. Another common outcome found was that expanded workforce positions increased preventative dental services for community members. The implementation of dental therapists increased knowledge and awareness about oral health, what causes dental diseases and methods to prevent tooth decay. Dental Health Aide Therapists aid in changing individual attitudes to value preventive dental care and education of residents [14].

## Discussion

Overall, limited available evidence has suggested that AI/AN identity is an important aspect of routine and accessible oral healthcare. There are unique barriers and motivations that personnel in the oral health workforce face, let alone the distinctiveness of serving AI/AN communities. In the 1960s, the Indian Health Service established the Community Health Aide Program (CHAP) in Alaska in response to a Tuberculosis outbreak. This included the Alaska CHAP program being recognized and funded by Congress. Since then, the program has flourished and has increased culturally appropriate access to behavioral, primary, and oral health care. In 2010, the Indian Health Care Improvement Act (IHCIA) was amended to authorize the Indian Health Service (IHS) to create a national CHAP, expanding to the lower 48 states. The significance and implications of the national CHAP program include the infrastructure needed to increase the number of AI/AN providers serving AI/AN communities while addressing the social determinants of care through accessible education, cultural and community connectedness through community-based care and outreach. While the term DHAs/DHAT and health aides in general as discussed in this paper detail the provider and various scopes of practice under the federal CHAP program, dental therapy in the general sense has grown more popular in recent years. This oral health care provider has been suggested as a solution to providing more routine and accessible care to underserved and minority populations [15, 16]. The dental therapy movement aims to provide communities with practitioners who can provide primary preventive and restorative care [16]. Findings from this review suggests that dental therapists not only are capable of providing vital routine care but many are also community members, increasing cultural knowledge amongst providers and providing local employment and furthering career opportunities.

AI/AN populations are at higher risk for severe dental disease, and more specifically a disproportionate prevalence of dental caries [17], especially when compared to the general population [18, 19]. Due to historical and ongoing systemic injustices, AI/AN populations do not have access to timely and quality oral health services. It has been suggested that despite rigorous recruitment initiatives and financial incentives, the IHS and tribes continue to struggle with attracting dentists [17]. Alarmingly, due to the substantial burden of disease among this population, even if all available IHS dental positions serving AI/AN communities were filled, the dentist to population ratio could still be insufficient to providing care for all [20]. This provides further justification for the need to expand the dental workforce, especially for rural and minority populations. One study found that 78% of practicing dental therapists in Alaska practiced in their own village or region of origin, and 87% are of AI/AN descent, with a retention rate for dental therapists in Alaska over 11 years to be 81% [21, 22].

As evidenced in the NIH Report titled “Oral Health in America: Advances and Challenges,” the social and demographic profile of the oral health workforce is not reflective of the nation’s population[2]. The report also emphasized diversifying the “jobs”of oral health professionals. Our findings agree with this call to action because establishing diverse workforce positions has been suggested to increase access to oral health care for many minority and low-income communities. We recently published actionable oral health policy and improvement strategies for the state of Oklahoma [23]. At the national scale, advances in oral health policies are a vital component of increasing access to oral health care for many communities. Recommendations to support workforce deficiencies include increasing supporting I/T/U (Indian Health Service, Tribal, Urban) representation on dental boards throughout the nation, as well as advocacy for the continued and expanded recruitment and retention of oral health care professionals within I/T/U health systems [23]. Further recommendations include indigenizing curriculum so that it is culturally relevant to communities, and establishing the infrastructure to recruit and train local community members for oral health professions. This approach is central to the CHAP program mentioned above, a program designed for tribes by tribes.

Systemic changes to the health care system to meet the demand for safe, quality, and affordable care for communities may require a mental model shift to make space for innovation. A variety of historical, cultural, regulatory, and policy barriers have limited ability of allied professionals to contribute to widespread and meaningful change. Organized medicine and dentistry have often challenged expanding the scope of practice for other allied and mid-level providers. Advocacy efforts are happening to support evidenced-based policies to meet the needs of underserved communities. Specifically, the 2022 National Indian Health Board’s Legislative and Policy Agenda includes requesting Congressional action to amend the Indian Health Care Improvement Act (IHCIA) to remove the state approval requirement for Tribal DHATs under the CHAP [24]. This requirement limits Tribes wanting to utilize DHATs under CHAP to those in states that license dental therapists. This provision raises a barrier between Tribes and oral health care services. However, many Tribes have begun actively engaging with states to ensure Tribes can employ dental therapists and have their services reimbursed by state Medicaid programs. Additionally, there is active advocacy to increase funding for IHS to continue CHAP expansion, funding for CHAP education and certification programs, and for the IHS scholarship and Loan Forgiveness program to include CHAP providers as eligible.

Future community-based research should investigate the pathway to oral health workforce positions among AI/AN communities. It is possible that recruitment initiatives and oral health career exploration programs tailored towards the culture and understanding of AI/AN community needs may aid in increasing AI/AN representation within the oral health workforce. Further, more research should be conducted exploring the impact that an expanded oral health workforce has on health outcomes and the social determinants of health among AI/AN communities.

Limitations to the studies include that most were retrospective observational studies, for which missing data is inevitable. More specifically, there are a lack of race and ethnicity variables, and other demographic variables of interest such as sex/gender, amount of time in the position, and salary of the oral health workforce positions to analyze. Most studies included did not have strict definitions of the workforce positions in order to reduce the risk of possible bias. Most studies had very small samples sizes due to the prevalence of oral health workforce positions in general, and especially among AI/AN community members.

This review provides findings that expanded oral health positions aid in community members receiving more routine and preventative care and is an upstream public health approach that has diversified the dental workforce. These types of community engagement and multidisciplinary approaches are proven to allow for greater opportunities for community members to receive vital care.
